# Advances in neutrophil extracellular traps and ferroptosis in sepsis-induced cardiomyopathy

**DOI:** 10.3389/fimmu.2025.1590313

**Published:** 2025-04-28

**Authors:** Man Zeng, Yuying Niu, Jiahao Huang, Liehua Deng

**Affiliations:** Department of Critical Care Medicine, Affiliated Hospital of Guangdong Medical University, Zhanjiang, China

**Keywords:** sepsis-induced cardiomyopathy, ferroptosis, neutrophil extracellular traps, neutrophil, programmed cell death

## Abstract

Sepsis-induced cardiomyopathy is a reversible non-ischemic acute cardiac dysfunction associated with sepsis. It is strongly associated with an abnormal immune response. It emerges as a vital threat to public health owing to its high mortality rate. However, the exact pathogenesis requires further investigation. In recent years, NETosis and ferroptosis, which are novel modes of programmed cell death, have been identified and found to play important roles in sepsis-related organ damage. This article outlines the mechanisms of these two modes of cell death, discusses the role of neutrophil extracellular traps in myocardial injury and the importance of ferroptosis in sepsis-induced cardiomyopathy, and reviews the potential interconnection between these two types of programmed cell death in sepsis-induced cardiomyopathy.

## 1 Introduction

Sepsis is a physiological, pathological, and biochemical disorder caused by infection, accompanied by multiorgan functional damage, inflammatory flare-ups and septic shock (1). Sepsis-induced cardiomyopathy (SIC) is a non-ischemic acute cardiac dysfunction associated with sepsis. It is associated with ventricular dilatation, reduced contractility and/or dysfunction, and decreased volumetric perfusion response (2). Myocardial dysfunction due to sepsis poses a risk that greatly increases mortality (3). The heart, as a pump organ, plays an important role in the pathophysiology of sepsis. In a retrospective study, it was found that cardiovascular and pulmonary injuries were dominant in sepsis induced organ dysfunction. And even after recovery from cardiomyopathy, SIC may continue to affect the patient’s body system (4, 5). Therefore, further exploration of the pathogenesis of SIC plays an important role in improving patient prognosis.

As the pathophysiology of sepsis is being increasingly studied, dysregulation of immune and non-immune cell death processes and mitochondrial dysfunction are being revealed in the context of sepsis-related organ damage (6). Numerous studies have shown that in addition to pyroptosis, programmed cell death (PCD) such as autophagy, apoptosis, NETosis, necrotic apoptosis, and ferroptosis are also involved in the development of organ damage in sepsis (7, 8). These new ideas may contribute to the development of novel therapeutic strategies for SIC.

In this paper, a brief overview of the mechanisms of SIC and the process of occurrence between the two types of programmed cell death, NETosis and ferroptosis, are described. The roles of these two types of cell death in the pathogenesis of SIC and myocardial injury as well as their possible interrelationships are also discussed.

## 2 Results

### 2.1 Pathogenesis of sepsis-induced cardiomyopathy

**Figure 1:**
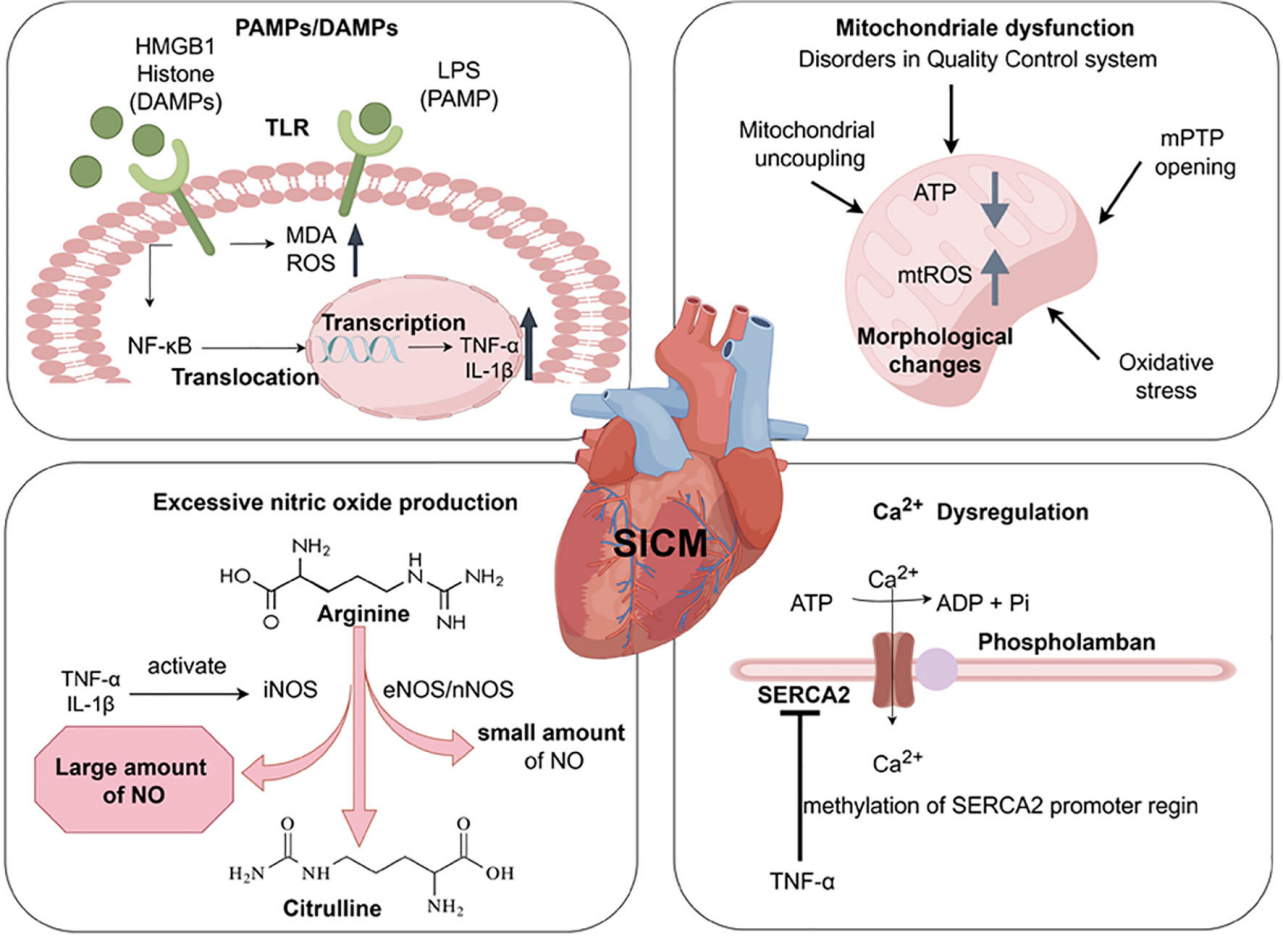
Pathogenesis of Sepsis-induced Cardiomyopathy. The release of LPS, HMGB1, and histone activates Toll-like receptors, increases the level of MDA and ROS. Through the (TLR)4/NF-κB pathway, chromosomal translocation occurs and inflammatory cytokines (TNF-α, IL-1β) are produced. These produced inflammatory cytokines activate nitric oxide synthase (including iNOS, eNOS, nNOS). It promotes the excessive production of NO during the conversion of arginine to citrulline, leading to cardiac dysfunction. The upregulation of inflammation leads to the occurrence of some dysfunctions in mitochondria, such as disorders in the quality control system, Mitochondrial uncoupling, Oxidative Stress, mPTP anomalous opening. These changes lead to structural changes in mitochondria (swelling, vesicle formation, abnormal cristae), ATP depletion, and an increase in mitochondrial reactive oxygen species (mtROS). TNF-α can downregulate the expression of SERCA2 in the sarcoplasmic reticulum, leading to reduced Ca^2+^ reabsorption in the sarcoplasmic reticulum. Created with www.figdraw.com.

SIC is a key feature of sepsis-related cardiovascular failure (2). Endothelial disruption and uneven distribution of blood flow occur in the microcirculation during sepsis. In sepsis that brings local ischemia, the distribution of coronary artery blood flow to the heart is uneven, but it does not induce significant cellular ischemia in the heart (9). The existing studies mainly involve dysregulation of the inflammatory response, nitric oxide (NO) production, mitochondrial dysfunction and abnormal Ca^2+^ regulation **Figure 1**.

The innate immune system plays a crucial role in the patient’s first line of defense against microbial invasion. The innate immune system is activated by pathogen-associated molecular patterns (PAMPs) (e.g., Bacterial lipopolysaccharide (LPS), viral double-stranded RNA) and damage-associated molecular patterns (DAMPs) (e.g., high mobility group protein B1 (HMGB1), extracellular histones, mtDNA, IL-1α/IL-33) via pattern recognition receptors (PRRs), which trigger inflammation (10–12). In addition, specific pathways activated by different PAMPs and DAMPs may lead to different outcomes of organ damage. For example, the activation of different TLRs may lead to different inflammatory features, which may affect the degree of organ damage (13). In the study of the pathogenesis of acute kidney injury, PAMPs and DAMPs can bind to TLR4, and the expression of TLR4 increases with kidney injury and/or infection (14). DAMPs mediated TLR4 signaling also plays a central role in acute lung injury (ALI) (15). Changes in intestinal permeability also may lead to the transfer of PAMPs and DAMPs into the systemic circulation, exacerbating inflammatory responses and causing organ dysfunction (16). During the development of SIC, PAMPs can be recognized. The Toll-like receptor family of proteins act as an innate immune response as a first line of defense against infection in sepsis (17). When SIC occurs, the released endotoxins activate Toll like receptors and produce pro-inflammatory cytokine (TNF-α, IL-1β, etc.) through the (TLR)4/NF-κB pathway. This directly affects the contractility of cardiomyocytes, leading to increased myocardial depression (2). Secondly, TLR2-5, TLR7, and TLR9, which are activated by inflammatory factors and NF-κB, are associated with septic cardiac dysfunction (18, 19). TLR2–5 are highly expressed in the myocardium (20). TLR2 also recognizes lipoproteins and peptidoglycans from Gram-positive bacteria, giving rise to myocardial damage. Whereas TLR2 and 4 can affect myocardial contractile function in sepsis (21). In addition, endotoxin induces an increase in malondialdehyde (MDA) levels, reactive oxygen species (ROS) production, and sarcoplasmic reticulum Ca^2+^ leakage through activation of TLR4 (22, 23). DAMPs include HMGB1, extracellular cold-inducible RNA-binding protein (eCIRP), adenosine triphosphate (ATP), and histones (24). It has been confirmed that HMGB1 and histones play an important role in cardiotoxicity in sepsis (13). Upon release, HMGB1 binds to TLR4 causing an increase in intracellular ROS and mediates Ca^2+^ release from the sarcoplasmic reticulum. It promotes cardiac inflammatory injury, cardiac regeneration and remodeling (25). At the same time, histones released from the myocardium can be cytotoxic to cardiomyocytes through TLR4- and NF-kB-independent signaling (26). In contrast, extracellular histones released from neutrophils mediated by complement C5a activate TLR2–4 and TLR9, affecting Ca^2+^ homeostasis in cardiomyocytes and leading to cardiomyocyte damage (27).

In advanced sepsis cardiac dysfunction, inflammatory cytokines (IL-1β, TNF-α) activated by increasing the inducible nitric oxide synthase (iNOS), which further sustains the production of excess NO (28). Meanwhile, small amounts of NO produced by endothelial (eNOS) and neuronal (nNOS) nitric oxide synthase (29). Many studies have suggested that in the late stage of sepsis with cardiac dysfunction, changes in ventricular preload/afterload, downregulation of β-adrenergic receptors, attenuated myofilament Ca^2+^ response, and mitochondrial dysfunction can all contribute to NO production through increased iNOS expression (2).

Mitochondrial dysfunction also promotes SIC, with the main changes about the disorders in mitochondrial quality control system, structural changes (swelling, vesicle formation, cristae abnormalities), mitochondrial DNA damage, and mitochondrial uncoupling (2, 30). LPS also promotes cardiac damage from oxidative stress in cardiac mitochondria, and brings to excessive mitochondrial uncoupling through uncoupling proteins (UCPs). These further results in ATP depletion and myocardial cell death (31, 32). During the occurrence of SIC, Ca^2+^ overload also induces abnormal opening of the mitochondrial permeability transition pore (mPTP). It causes mitochondrial membrane potential disorder, mitochondrial outer membrane swelling and rupture (33).

Sarcoplasmic reticulum Ca^2+^-ATPase (SERCA2) is a key regulator of Ca^2+^ in cardiomyocytes. TNF-α released during sepsis enhances methylation of the SERCA2 promoter region, leading to down-regulation of SERCA2 expression. It reduces sarcoplasmic reticulum Ca^2+^ reabsorption and brings disorders to ventricular diastolic function (2, 34).

### 2.2 Formation of neutrophils extracellular traps

Neutrophils, as immune cells, are the line of defense against infection and play a significant role in limiting the expansion and spread of bacterial and viral infections (35). When the organism is infected, a large number of bone marrow neutrophils are released into the circulation to play a phagocytic role or release inflammatory cytokines, reactive oxygen species, and other antibacterial substances (35). In addition to traditional antibacterial mechanisms, there is a significant correlation between specific neutrophil functional phenotypes and the severity of sepsis (36). During sepsis, the bone marrow releases a large amount of immature granulocyte (IGs) through emergency granulopoiesis (37), such as *CEACAM8^+^* Neu, *S100A8/9hi*Neu, *IL1R2^+^* Neu, *PADI4^+^* Neu, *MPO^+^* Neu and cycling *MK167^+^CYP1B1^+^* Neu (38). In the early stage of sepsis, CD64_pos_ and CD16_dim_ IGs significantly increase with the severity of sepsis, which can predict the severity of early sepsis and provide guidance for further treatment (39, 40). In the late stage and recovery period of sepsis, studies have found that infiltrating neutrophils have immunomodulatory effects. Neutrophil subpopulations with high expression of PD-L1 can exert immunosuppressive effects in direct contact mode (41). Meanwhile, *CD66b^+^* Neu can inhibit the proliferation and activation of CD4^+^T cells and produce the effection of immunosuppressive (38).The marker for neutrophil activation and release of neutrophil extracellular traps (NETs) is S100A8/A9, which is mainly released by polymorphonuclear neutrophils (PMNs) (42).S100A8/A9 can not only inhibits mitochondrial function as the main regulator of myocardial cell death (43), but also promotes the secretion of interleukin-1 β, leading to stimulates granulopoiesis (44). Some studies have also found that S100A8 is an independent risk factor for poor prognosis in sepsis patients (45).

**Figure 2:**
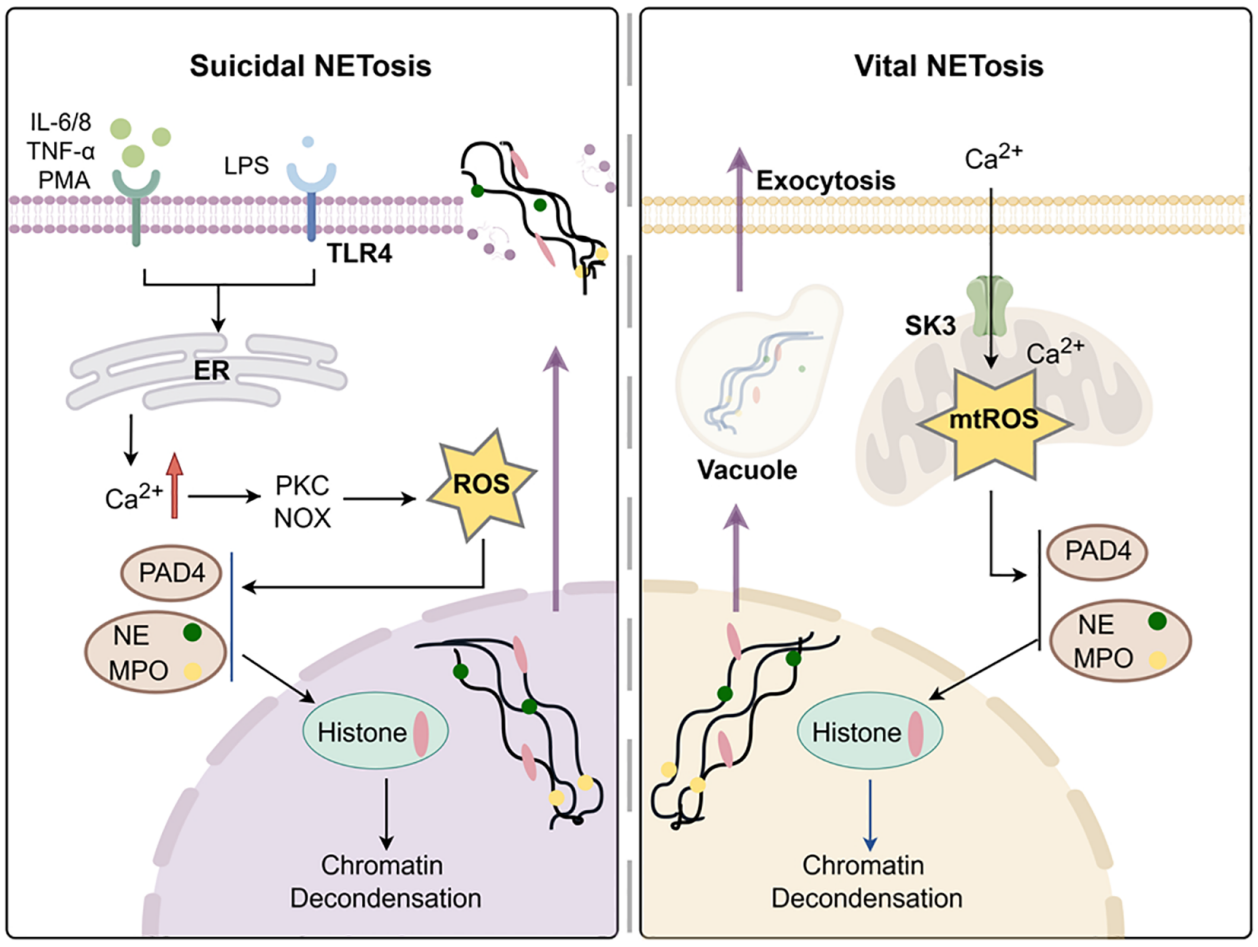
Formation of neutrophils extracellular traps. Created with www.figdraw.com.

In 1996, a novel type of neutrophil death independent of apoptosis and necrosis was first discovered when neutrophils were stimulated (46). This kind of neutrophil death is accompanied by depolymerizing chromatin, dissolving the nuclear membrane and releasing chromatin encapsulated in granulin (46). In 2004, Brinkmann’s group defined extracellular structures consisting of DNA, histones and granzymes (e.g. neutrophil elastase) as NETs (47). NETosis is usually categorized into two different types - suicidal NETosis that relies on NADPH oxidase (NOX) and vital NETosis that is NOX independent (48) **Figure 2**.

Suicidal NETosis is formed during active cell death, which requires membrane rupture and loss of neutrophil function, which is dependent on NOX for the production of ROS (48). During the suicidal NETosis, different neutrophil receptors are activated. This triggers the release of endoplasmic reticulum (ER) calcium stores, thus leading to an increase in cytoplasmic Ca^2+^ (49). Excess Ca^2+^ accumulation activates protein kinase C (PKC), phosphorylation of gp91phox and assembly of functional NADPH oxidase, ultimately resulting in the generation of ROS (50). Excessive ROS causes neutrophil elastase (NE) and myeloperoxidase (MPO) to escape from azurophilic granules and translocate to the nucleus, where they partially degrades specific histones, promoting chromatin decondensation (51). Concurrently, Ca^2+^ acts as cofactors for peptidyl arginase deaminase 4 (PAD4), which promotes chromatin depolymerization by facilitating the loss of the positive charge required for histone-DNA interaction (52, 53).

Finally, the dissociated chromatin is released into the extracellular space through membrane pores and accompanied with cell death (54). However, in the study of healthy donor neutrophils, unlike oxidase-independent NETosis induced by Ca^2+^, calcium ionophores can also induce mtROS formation, thereby stimulating NOX to produce NETosis (55).

In 2012, rapid release of NETs from living PMNs *in vivo* was first observed, which is distinguished from the traditional suicidal NETosis (56). This type of NETosis, without cell death, is called, vital NETosis (48). Unlike suicide NETosis, which takes up to 3–4 hours to occur through NOX dependence, the vital NETosis only takes 5–60 minutes and is mainly induced by Ca^2+^ carriers to appear independently of NOX (57). The characteristic of important NETosis is the opening of mPTP. It activates SK3 channels through Ca^2+^ in mitochondria, generates mtROS to catalyze histone citrullination and forms DNA vesicles (55, 58). These DNA vesicles sprout from the nuclear membrane, pass through the cytoplasm, and fuse with the plasma membrane to be released outside the cell without the need for membrane perforation (59).

NETosis stimulated by different agonists occurs through different pathways (60). Interestingly, all NETs generated by stimuli are mainly composed of chromosomal DNA and can degrade proteins and kill bacteria (60, 61). Compared to the nonphysiological NOX-dependent agonist PMA, the sepsis experimental model stimulated by LPS found in gram-negative bacterial infections more closely simulated *in vivo* conditions (62). It has been demonstrated that LPS activates the TLR4-JNK activation axis in a dose-dependent manner to initiate NOX-dependent suicidal NETosis, which differs from PMA (63).

### 2.3 Vital role of NETs in myocardial injury

NETs are key components of the immune response under various pathological conditions. In 2004, it was found that during the fusion of antibacterial granules and phagosomes in neutrophils, NETs are released to clear pathogens and protect the organism (47). However, after excessive production of NETs by neutrophils, tissue damage is induced in sepsis (64). This increases various inflammatory factors, cell death in different organs and aggravates the progression of the disease (65).

NETs are involved in various myocardial injury diseases by causing microvascular dysfunction, impaired cardiac contractile function, myocardial fibrosis, and inflammatory responses. Firstly, with the intensive study of microvascular dysfunction, it has been found that oxidative stress induced by the inflammatory response to ROS accumulation can drive coronary microvascular dysfunction (66). The accumulation of NETs in small blood vessels increases thrombus formation. This leads to a scarcity of myocardial capillary density, a decrease in myocardial blood flow, and an increase in the expression of ischemic markers (67). These factors trigger restrictive myocardial ischemia, cardiomyocyte death, and ultimately cardiac hypertrophic remodeling (68). NETs are equally involved in myocardial microcirculatory obstruction induced by ischemia-reperfusion injury. Further studies demonstrated that the combination of the inhibitor of NETs (DNase I) and recombinant tissue-type plasminogen activator (rt-PA) exerted a protective effect against myocardial ischemia-reperfusion (69).

Additionally, myocardial dysfunction is characterized by transient biventricular impairment of intrinsic myocardial contractility. It has shown that extracellular guanidine histones driven by NETs can induce alterations in intracellular mitochondrial respiration. This affects the contractile performance of cardiomyocytes (70), e.g. reduced left ventricular ejection fraction (LVEF) (67). Reduced LVEF also occurs in sepsis induced myocardial injury, but further research is still needed to confirm whether sepsis induced myocardial injury worsens in the occurrence of NETosis (71). Nevertheless, several factors, including the release of transforming growth factor (TGF) from NETosis, stimulate cardiac fibrosis. These bring to the development of ischemic cardiomyopathy (ICM) and dilated cardiomyopathy (DCM) (72).

Finally, NETs, with highly pro-inflammatory properties, are observed to form and induce the occurrence of myocarditis (73), which is induced by cytokine mediator factor (MK) (74). It is worth noting that the main extracellular structures of NETs include DNA, histones, and neutrophil elastases. However, in the progression of myocardial injury, it is still unclear which component plays a key role in the pathogenesis or the synergistic effect of multiple components.

### 2.4 Mechanisms of ferroptosis

**Figure 3:**
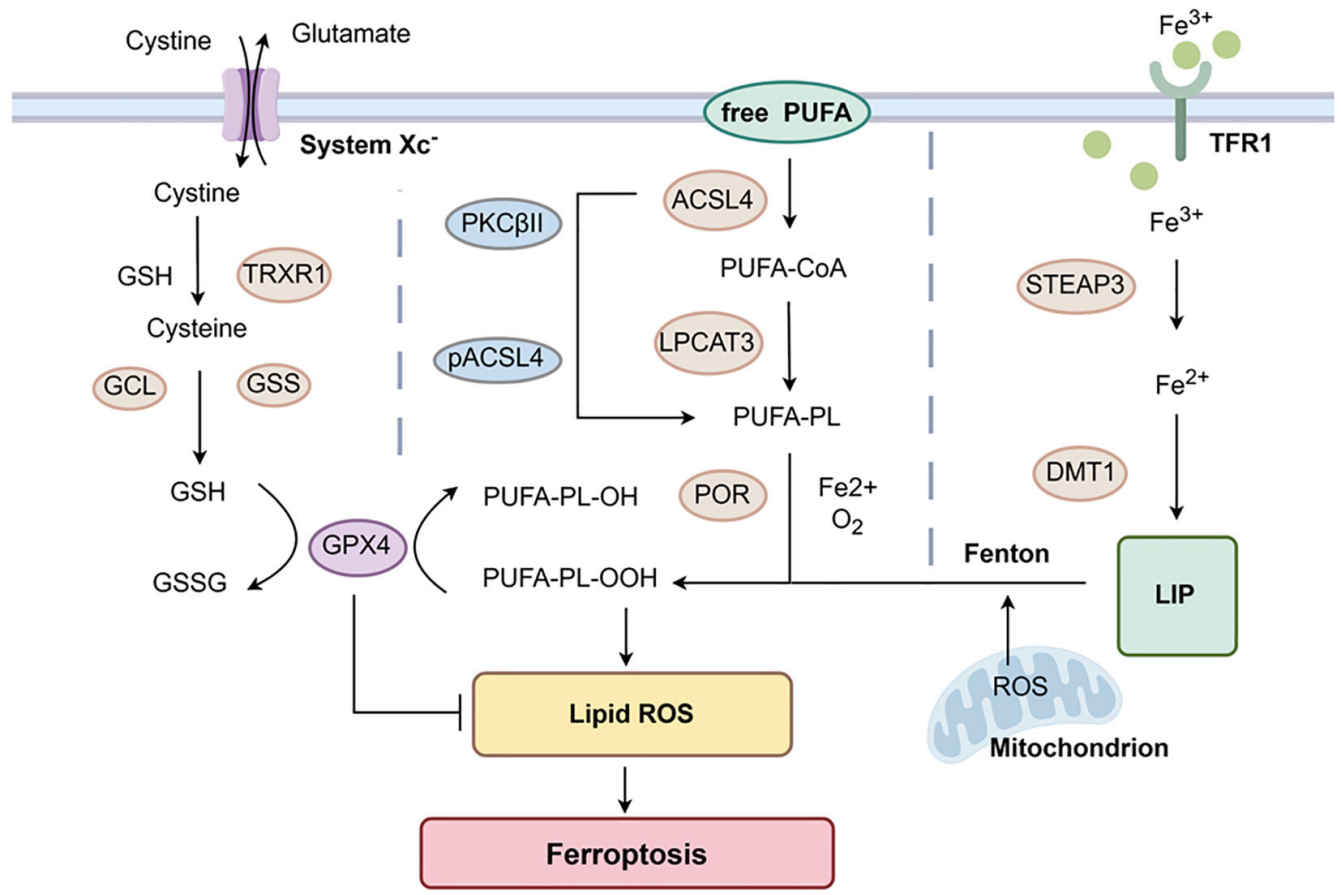
Mechanisms of Ferroptosis. GPX4-mediated ferroptosis control axis requires system Xc^-^, GSH, and TRXR1 to reduce cystine to cysteine. GSH also participates in the reduction of PUFA-PL-OOH mediated by GPX4, inhibiting the occurrence of ferroptosis. Free PUFAs change into PUFA-PL under the action of ACSL4 and LPCAT3 in unison. This process is further activated by phosphorylation of PKCβII. The oxidation reaction involving Fe^2+^ generates PUFA-PL-OOH, which undergoes lipid peroxidation and induces ferroptosis. TFR1 can help Fe^3+^ get into cells. Under the action of STEAP3, Fe^3+^ is reduced to Fe^2+^. These excess Fe^2+^ are transported to the LIP via DMT1, participating in the initiation of the Fenton reaction to rapidly generate PUFA-PL-OOH and induce ferroptosis. Created with www.figdraw.com.

Ferroptosis is characterized by lipid peroxidation, which is distinct from apoptosis, necrosis, and autophagy. Its main morphological changes include the condensation of mitochondrial membrane density, the reduction or disappearance of mitochondrial cristae, and the rupture of the outer mitochondrial membrane (75). Ferroptosis was first identified in 2001 as known as oxidative glutamate toxicity. It is caused by exogenous glutamate inhibiting cysteine uptake through cysteine/glutamate reverse transporters, leading to depletion of glutathione (76). In 2012, Dixon’s group first named this cell death as ferroptosis while studying the mechanism in an oncogenic RAS mutant cell line killed by erastin (77). These following mechanisms are primarily involved in ferroptosis **Figure 3**.

Glutathione peroxidase 4 (GPX4)-mediated cysteine metabolism is mainly involved in ferroptosis. It was found that the cystine/glutamate reverse transporters (system Xc^-^) present on the cell surface act as reverse transporter, driving cysteine and glutamate to enter and exit the cell in a 1:1 ratio based on concentration gradients (78). Cysteine is transported into the cell and reduced to cysteine by GSH or thioredoxin reductase 1 (TRXR1), which provides the raw material for intracellular GSH synthesis (79–81). Simultaneously, GSH is produced in an enzymatic reaction with GCL (glutamate-cysteine ligase) and GSS (glutathione synthetase) (82). GPX4 is a selenoprotein that can degrade PUFA-PL-OOH to PUFA-PL-OH through glutathione (GSH) and reduce the accumulation of lipid peroxides (83, 84). GSH is also an essential reaction substrate for the degradation of LPO by GPX4 (79). After inhibiting the activity of system Xc^-^, it affects the absorption of cysteine and the synthesis of GSH. This decreases the activity of GPX4, cellular antioxidant capacity and lipid ROS accumulation, and the outcome of oxidative damage and ferroptosis (85).

Unrestricted lipid peroxidation occurs within cells. Acyl coenzyme A synthetase long-chain family member 4 (ACSL4) and lysophosphatidylcholine acyltransferase 3 (LPCAT3) are also the key proteins in the development of ferroptosis (86, 87). Polyunsaturated fatty acids (PUFAs) are components of cell membranes that are highly susceptible to peroxidation. It was shown that free PUFAs are incorporated into membrane lipids by activation of ACSL4 and form acyl-CoA with coenzyme A (CoA). Subsequently, with the effect of lysophosphatidylcholine acyltransferase 3 (LPCAT3), acyl-CoA can be re-esterified in phospholipids to form PUFA-PL (88, 89). ACSL4 can also be phosphorylated by PKCβII to further activate this process (80). Then PUFA-PL is oxidized by labile Fe^2+^ and Fe^2+^-dependent enzymes to PUFA-PL-OOH, which ultimately undergoes lipid peroxidation and ferroptosis (88).

Cellular toxicity by accumulation of Fe^2+^: Transferrin (Tf) safely delivers iron through circulation to cells (90). The extracellular Fe^3+^ bound to transferrin receptor (TfR)1 enters the cell by endocytosis and is reduced to Fe^2+^ under the action of iron oxide reductase (STEAP3) (91). NRAMP2 is also an iron transport protein first discovered by Nancy and later renamed DMT1 (92, 93). The reduced Fe^2+^ is transported to the labile iron pool (LIP) via DMT1 and then translocated to the mitochondria to participate in generation of ROS. Upon accumulation of excess Fe^2+^, the Fenton reaction is initiated to rapidly generate PUFA-PL-OOH to induce ferroptosis (80, 88, 91). In the last few years, non-GPX4-dependent ferroptosis has also been found to occur, e.g., ferroptosis suppressor protein 1 (FSP1) was identified as a key component of the non-mitochondrial coenzyme Q (CoQ10) antioxidant system. It plays a parallel role to the classical glutathione-dependent GPX4 pathway (94).

In addition to ferroptosis, iron overload may also participate in other inflammatory cell death mechanisms to cause disease. Free Fe^2+^and Fe^3+^are easily converted to each other and catalyze the Fenton reaction, which produces oxygen free radicals. The accumulation of these ROS can further lead to cellular damage (95). Iron overload enhances the ROS signaling pathway induced by carbonyl cyanide m-chlorophenyl hydrazone (CCCP), which can amplify ROS signals to drive the occurrence of cell pyroptosis (96, 97). Excessive iron ions can not only disrupt cellular iron homeostasis, leading to oxidative stress and apoptosis (98), but also induce apoptosis by triggering endoplasmic reticulum (ER) stress, resulting in mitochondrial dysfunction (99). In addition, studies have shown that iron overload induced ROS can promote necrotic apoptosis of osteoblasts (100). In the study of acute kidney injury, it has also been confirmed that there is an interactive relationship between ferroptosis and necrotic apoptosis, and ferroptosis may become a driving factor for necrotic apoptosis (101). The interconnection of these modes of death may further deepen our understanding of the disease.

### 2.5 Role of ferroptosis in the pathogenesis of Sepsis-induced cardiomyopathy

Ferroptosis has emerged as a hot research topic in the development of diseases. In the early stages of the immune response, it has been reported that ferroptosis helps macrophages inhibit intracellular bacteria by reversing the input of ferrous iron into bacterial vacuoles through iron transporters and acts as a protector of the organism (102). However, in Mycobacterium tuberculosis-infected cells, ferroptosis similarly promotes cell death and tissue necrosis (103). Viral infection usually leads to the occurrence of viral myocarditis (104). With the development of disease progresses, some patients may experience myocardial damage (105). It was found that TRIM29 (Tripartite motif 29) can regulate alveolar macrophage activation to mitigate bacteria-induced sepsis (106) and controls viral myocarditis by modulating protein kinase RNA-like endoplasmic reticulum kinase (PERK)-mediated ER stress and ROS responses (107). Simultaneously, the expression of PERK and SLC7A11 is positively correlated and inhibits ferroptosis (108). TRIM18 (Tripartite motif 18) is also one of the negative regulators of immune response. It can control viral myocarditis by recruiting protein phosphatase 1A to regulate TANK binding kinase 1 (TBK1)-mediated immune responses (109). In addition, the regulation of TBK1 can also induce ferroptosis (110). Similarly, PARP9 (poly (ADP-ribose) polymerase 9) manages viral myocarditis by engaging the PI3K/AKT pathway to drive type I interferon responses (111). A series of studies have found the involvement of PI3K/AKT in the mechanism of ferroptosis (112). Therefore, TRIM29, TRIM18, and PARP9 may regulate ferroptosis to manage cardiomyopathy.

The role of ferroptosis in sepsis-induced cardiomyopathy has received considerable attention. Lipid peroxidation is an important step in ferroptosis. It has been shown that, in a mouse model of LPS induced SIC, the increasing of ICA69 affects STING signaling, thereby leading to the generation of lipid peroxidation in cardiomyocyte. This ultimately results in ferroptosis and cardiac injury (113). Meanwhile, miR-130b-3p was found to significantly upregulate the expression of GPX4 and inhibit the activity of ACSL4, which reduces the production of lipid ROS and ferroptosis and improves the cardiac function of mice (114). By upregulating the Sirt1/Nrf2 pathway, it can also reform myocardial ferroptosis caused by iron metabolism imbalance and lipid peroxidation damage (115).

An imbalance in iron homeostasis is also a prominent feature of ferroptosis. After LPS stimulation, myocardial cells increase the expression of nuclear receptor coactivator 4 (NCOA4), which in turn participates in ferritin autophagy and releases a large amount of Fe^2+^. Excessive accumulation of Fe^2+^ in the cytoplasm enters mitochondria, producing mitochondrial ROS and affecting the occurrence of ferroptosis (116). Exogenous lipid carrier protein 2 (LCN2) can also increase the intracellular LIP in cardiomyocytes, resulting in cellular ferroptosis (117).

GPX4-mediated cysteine metabolism is also involved in sepsis-associated myocardial injury. MiR-31-5p attenuates LPS-induced cardiomyocyte ferroptosis by regulating SLC7A11 deubiquitination. This provides new therapeutic ideas for the treatment of SIC (118). N6-methyladenosine writer METTL3 can also accelerate the sepsis-induced myocardial injury by m6A modification of SLC7A11 via YTHDF2 pathway (119, 120).

Likewise, iron metabolism was found to crosstalk with the glutathione cycle, inducing ferroptosis in cardiomyocytes. Cardiac-specific knockdown of ferritin H (FTH) decreased SLC7A11 in cardiomyocytes, reduced GSH levels and led to dysregulation of iron homeostasis and myocardial oxidative stress injury (121).

### 2.6 NETs interact with ferroptosis to accelerate sepsis-induced cardiomyopathy

In previous studies, neutrophils have been found to be involved in the pathogenesis of various systemic diseases by inducing ferroptosis through a novel pathway, such as NETosis. During abdominal aortic aneurysm (AAA) formation, NETs affect the stability of the mitochondrial carrier protein SLC25A11. This brings to depletion of mitoGSH, and promote ferroptosis in smooth muscle cells (SMCs) (122). Inhibition of the PI3K/AKT pathway by NETs also achieves this effect (122, 123). Inhibition of NETs formation was also demonstrated in 2023 to attenuate intestinal endothelial ferroptosis by improving Fundc1-dependent mitochondrial autophagy. In 2023, it was also confirmed that targeting NETs may be a promising approach for treating intestinal microcirculation dysfunction, since it modulates Fundc1-dependent mitochondrial autophagy to regulate intestinal endothelial ferroptosis (124).

Several recent studies have shown that NETs play a close synergistic role in the development of sepsis and ferroptosis. When sepsis occurs, the overproduction of NETs, which act as DAMPs, causes an inflammatory response, increases cellular iron transport and uptake, and makes cells more susceptible to ferroptosis, and consequently brings to functional impairment of various organs.

In the pathogenesis of sepsis associated acute lung injury, NETs can promote m6A modification and mitochondrial metabolic reprogramming of hypoxia inducible factor-1 α (HIF-1 α) induced by METTL3, leading to ferroptosis of alveolar epithelial cells and causing lung injury (125, 126). It has been found that mesenchymal stem cells (MSCs) not only inhibit the formation of NETs through the MEK/ERK signaling pathway, but also attenuate the ferroptosis of lung tissue in sepsis-induced ALI (127). Further studies revealed that inhibition of NETs production also attenuates ferroptosis and plays an important role in ALI by maintaining the normal SDC-1/HS/HGF/cMET signaling pathway (128). In addition, redox regulators and ferroptosis inhibitors (such as FS-1, Lpx-1, and DFO) can inhibit heme induced ferroptosis, and platelet-mediated NETosis is prone to form pulmonary thrombosis. They may be a potential adjunctive therapy for clinical complications associated with respiratory distress (129).

Moreover, ACSL4 knockdown significantly reduced lipid oxidation-induced ferroptosis in AKI model of mice (130). GPX4 has also been shown to be an important downstream mediator of HDAC3 (histone deacetylase 3) aberrations and renal ferroptosis during the AKI-CKD transition (131). Extracellular histones contribute to the development of acute kidney injury by directly releasing proinflammatory cytokines via TLR2/4. However, the source of these extracellular histones still needs to be explored whether they come from the components of NETs, and whether they can become a new way to induce ferroptosis (132).

NETs and ferroptosis may have a potential synergistic effect on SIC. It is characterized by sepsis-induced myocardial contractile dysfunction manifested by a reduced left ventricular ejection fraction. Interestingly, the accumulation of NETs negatively correlated with cardiac contractile function, highlighting the potential impact of NETosis on sepsis related cardiac injury (133). But further clinical studies are still lacking. It has been found that the ferroptosis related gene Mgst2 induces NOX-dependent NETosis and exacerbates the damage caused by cardiomyocytes, cardiac fibroblasts and endothelial cells (134). However, further exploration is needed to determine whether excessive NETs can drive ferroptosis in myocardial cells through related pathways in SIC. In addition to the possible association between NETs and ferroptosis in sepsis-induced cardiomyopathy, more attentions should be paid to the relationship between these two types of cell death and the studies in other sepsis related organ damage, such as the lungs and kidneys.

## 3 Discussion

After the body is infected and sepsis occurs, the outbreak of inflammation ultimately leads to systemic multi-organ dysfunction and hypotension (septic shock) (135). Based on the high mortality rate of sepsis-associated cardiac injuries in clinical practice, further investigation into the details of their pathogenesis is imminent (4). With further studies of programmed cell death in sepsis, NETosis and ferroptosis can provide new ideas for the treatment of sepsis-induced cardiomyopathy (7).

This article describes the formation of NETs and the mechanism of ferroptosis, summarizes the recent role of NETs in myocardial injury, and discusses the relevant mechanisms and roles of ferroptosis in the occurrence of SIC. After understanding the relationship between NETs and ferroptosis in various diseases, it was found that there are still many issues about the interaction between NETs and ferroptosis in SIC that need further exploration.

Above all, in the pathophysiological process of sepsis, there are many biomarkers that can mediate tissue damage, including pro-inflammatory cytokines, chemokines, and markers of neutrophil and monocyte activation (CD64, CD11b, TREM-1, etc.) (136). However, NETosis can occur through different pathways, and the ways in which NETosis is stimulated in sepsis still need to be explored. NETs can effectively trap bacteria in the circulation, during the occurrence of disease (137). In contrast, excessive neutrophil activation and release of NETs may have pro-inflammatory and procoagulant effects (137). Consequently, it is still difficult to control the content of NETs and balance their beneficial and harmful effects.

In addition, research on the mechanisms of ferroptosis in sepsis-associated heart is still in its preliminary stages. Further studies and experiments are needed to explore more pathways of ferroptosis in sepsis and the major signaling pathways in cardiac injury; however, the contribution of different organelles, including mitochondria, to ferroptosis in sepsis-induced cardiac injury is not fully understood. The relationship between NETs and ferroptosis still needs to be explored through clinical and mechanistic studies.

Finally, there are still no drugs approved in clinical therapy that specifically targets the formation of NETs and the occurrence of ferroptosis. It is worth noting that some laboratories have found that extracellular vesicles (MSC-EVs) derived from mesenchymal stem cells can prevent the formation of abdominal aortic aneurysms by inhibiting NET-induced ferroptosis (123). These MSC-EVs can similarly reduce NETs formation by restoring mitochondrial function, modulate ferroptosis and accelerate diabetic wound healing through inhibition of the PI3K/AKT pathway (138). Targeting the occurrence of NETs and ferroptosis through the combination of new biomaterials may become a new strategy for the treatment of sepsis-related organ damage.
